# Colloidal Templating in Catalyst Design for Thermocatalysis

**DOI:** 10.1021/jacs.4c07167

**Published:** 2024-08-05

**Authors:** Kang Rui Garrick Lim, Michael Aizenberg, Joanna Aizenberg

**Affiliations:** † Department of Chemistry and Chemical Biology, Harvard University, Cambridge, Massachusetts 02138, United States; ‡ John A. Paulson School of Engineering and Applied Sciences, Harvard University, Cambridge, Massachusetts 02138, United States

## Abstract

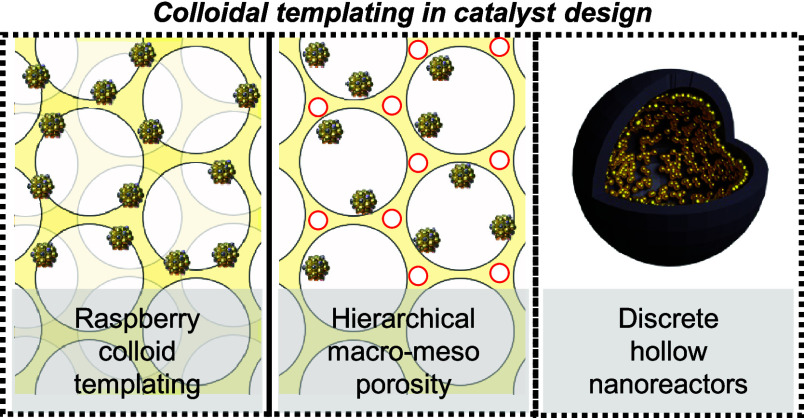

Conventional catalyst preparative methods commonly entail the impregnation, precipitation, and/or immobilization of nanoparticles on their supports. While convenient, such methods do not readily afford the ability to control collective ensemble-like nanoparticle properties, such as nanoparticle proximity, placement, and compartmentalization. In this Perspective, we illustrate how incorporating colloidal templating into catalyst design for thermocatalysis confers synthetic advantages to facilitate new catalytic investigations and augment catalytic performance, focusing on three colloid-templated catalyst structures: 3D macroporous structures, hierarchical macro-mesoporous structures, and discrete hollow nanoreactors. We outline how colloidal templating decouples the nanoparticle and support formation steps to devise modular catalyst platforms that can be flexibly tuned at different length scales. Of particular interest is the raspberry colloid templating (RCT) method which confers high thermomechanical stability by partially embedding nanoparticles within its support, while retaining high levels of reactant accessibility. We illustrate how the high modularity of the RCT approach allows one to independently control collective nanoparticle properties, such as nanoparticle proximity and localization, without concomitant changes to other catalytic descriptors that would otherwise confound analyses of their catalytic performance. We next discuss how colloidal templating can be employed to achieve spatially disparate active site functionalization while directing reactant transport within the catalyst structure to enhance selectivity in multistep catalytic cascades. Throughout this Perspective, we highlight developments in advanced characterization that interrogate transport phenomena and/or derive new insights into these catalyst structures. Finally, we offer our outlook on the future roles, applications, and challenges of colloidal templating in catalyst design for thermocatalysis.

## 1 Introduction

**Figure 1:**
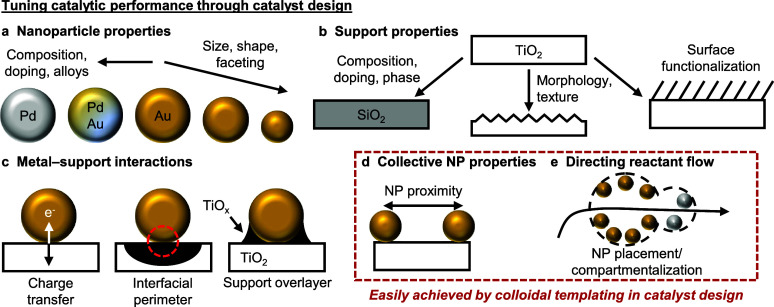
**Synthetic approaches to tune catalytic performance through catalyst design.** The catalytic performance of NP-supported catalysts can be tuned using conventional catalyst preparation methods through altering the (a) NP properties, (b) support properties, and/or (c) metal–support interactions. Additional means to influence catalytic performance include tuning (d) collective NP properties, such as NP proximity, placement, and compartmentalization, and (e) directional control over reactant flow. Control over (d) and (e) is challenging to achieve using conventional catalyst preparation methods but can be more easily tuned by incorporating colloidal templating into catalyst design. (a)–(c) adapted with permission from ref (4). Copyright 2019 Springer Nature.

Nanoparticle (NP)-supported catalysts comprise NPs stabilized on supports and constitute the majority of heterogeneous catalytic systems used industrially today.^1,2^ These catalysts are traditionally produced by the impregnation or precipitation of metal precursors on preformed supports or support precursors, and their catalytic performance is primarily tuned through the NP properties, support chemistry, and the interactions between them (Figure 1a–c).^3−6^ While the individual components (NPs and support) as well as their properties can be readily changed using these catalyst preparative methods, such methods often require additional synthetic design steps to control collective ensemble-like NP properties,^7−9^ such as NP proximity, (asymmetric) placement, and compartmentalization (Figure 1d), without concomitant changes to other catalytically relevant descriptors. For instance, NP size and proximity are intimately coupled together when precipitation and impregnation methods are used as the metal precursor concentration controls the nucleation of new NPs, thereby reducing NP proximity, but simultaneously induces the growth of existing NPs.^10,11^ Moreover, the support chemistry also influences the NP size during nucleation,^5^ consequently affecting catalytic performance.^12,13^ This high degree of NP–support interdependence in traditional catalyst synthesis, while convenient in many scenarios, does not easily permit the full decoupling of the NP and support properties for independent tuning and structure–property optimization. Separately, directing reactant flow is essential for controlling selectivity in multistep catalytic cascades,^14−17^ but remains challenging to achieve in fully open or morphologically isotropic support structures (i.e., spherical and flat supports), since such catalyst designs do not possess predefined ingress and egress points to prescribe directional reactant flow (Figure 1e).^18−21^

**Figure 2:**
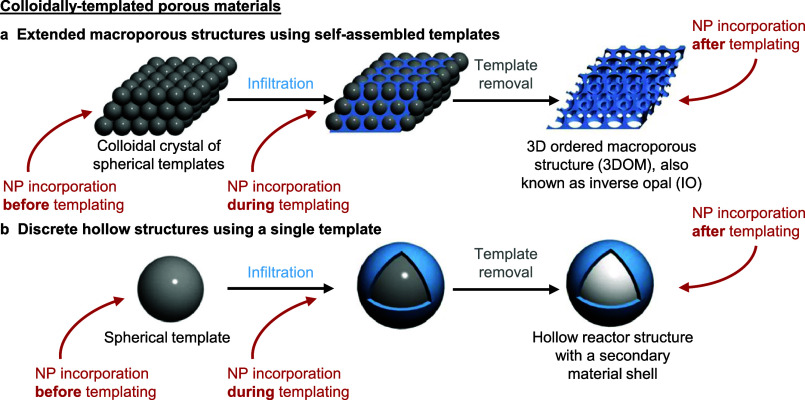
**Two general colloidal templating approaches to produce porous structures for catalysis.** Colloidal templating entails the (a) infiltration or (b) overgrowth of a second material, typically the catalytic support (blue), around (a) a colloidal crystal of self-assembled templates or (b) a discrete template (gray). Subsequent template removal reveals (a) an extended 3D macroporous structure or (b) a discrete hollow structure. In both types of colloidal templating, catalytically active NPs can be incorporated before, during, or after the colloidal templating process (red arrows). (a)–(b) adapted with permission from ref (28). Copyright 2012 American Chemical Society.

In this regard, incorporating colloidal templating into catalyst design allows one to exert greater isolated control over the individual components (NP or support)^22^ and collective NP properties,^23^ and/or realize the fabrication of anisotropic support architectures to direct and examine the effect of reactant flow within the catalyst.^24,25^ Colloidal templating describes the process of either infiltrating a close-packed arrangement of self-assembled colloidal templating particles (such as spheres, rods, *etc*.) collectively known as the colloidal crystal (Figure 2a), or the overgrowth of secondary material(s) around a single colloidal templating particle (Figure 2b), followed by the removal of the colloidal template(s), typically by combustion, dissolution, or etching.^26−28^ Note that the shape of the colloidal template is not restricted to only spheres: asymmetrically shaped templates and even a combination of templates of different sizes and shapes have been used together to direct transport in the final structure,^29−31^ which will be discussed in Sections 3 and 4. Concepts of colloidal templating first gained traction with the synthesis of mesoporous silicas (with 2–50 nm pores)^26^ and 3D ordered macroporous materials (3DOM, with >50 nm pores)^32−35^ to expand beyond the application scope of microporous zeolites (with much smaller <2 nm pores). While these 3DOM structures, also known as inverse opals (IOs), are more frequently utilized in the fields of photonics, sensing, metamaterials, and to some extent, photo-, electro-, and photoelectro-catalysis,^26,36−43^ growing interest and developments in colloidal NP synthesis^44,45^ have enabled the incorporation of catalytically active NPs in such colloid-templated structures for thermocatalysis.^22,24,25,46^

In this Perspective, we critically assess important recent progress in colloidal templating strategies applied in catalyst design for thermocatalysis, focusing on three NP-containing structures: extended 3D macroporous structures (Section 2), extended hierarchical 3D macro-mesoporous structures (Section 3), and discrete hollow nanoreactors (Section 4). *We highlight examples whereby colloidal templating is deliberately integrated into catalyst design to isolate and facilitate the unambiguous interrogation of collective NP properties, achieve spatially disparate active site compartmentalization, and/or direct reactant flow in multistep thermocatalytic cascades*—all of which we have identified as challenging to achieve using conventional precipitation and impregnation synthetic approaches for reasons described earlier. To complement these synthetic advances, we highlight developments in advanced characterization tools such as electron tomography,^23,47^ nuclear magnetic resonance (NMR) relaxation-exchange correlation,^24,48,49^ and single-particle tracking,^50−53^ all effectively applied in these catalytic structures in concert with computational simulations,^21,23^ to better understand fundamental mass transport phenomena and probe the catalyst structure. Finally, we offer our outlook on the anticipated future roles, developments, and challenges of colloidal templating in catalyst designs for thermocatalysis (Section 5). For conciseness, we will not discuss the more widely studied extended mesoporous catalyst structures without macropores (such as SBA-15 and MCM-41 mesoporous silicas), or microporous catalyst structures (such as zeolites and metal–organic frameworks). We refer readers to recent reviews^54−59^ that comprehensively evaluate these structures for heterogeneous catalysis. Moreover, while we center our discussions around colloidal templating in catalyst design for thermocatalysis, we acknowledge the closely related application of colloidal templating in photo-, electro-, and photoelectro-catalyst/electrode design and likewise refer readers to these complementary reviews.^60−64^

## 2 NP-Containing Extended Macroporous Structures

3D ordered macroporous (3DOM) structures, also known as inverse opal (IO) structures, initially garnered research attention in photonics due to their 3D structural periodicity.^32−35^ Given that even minor point or line defects can extensively propagate and considerably affect their optical properties,^65^ early efforts were focused on producing large area and crack-free structures.^66−69^ For instance, to minimize stress fracture arising from polycondensation-induced volume shrinkage of the infiltrated metal oxide sol–gel^70^ (e.g., silica, titania, both common catalytic supports) in the colloidal crystal during calcination to remove the templating colloids, our group reported an alternative approach of coassembling the templating colloids in the presence of the sol–gel precursor.^67^ Other approaches involve coassembling templating colloids with nanocrystals to form 3DOM/IO structures templated entirely using nanocrystals.^68,69,71,72^ The scalable wet chemical synthesis of high quality crack-free 3DOM/IO structures laid the foundation for incorporating catalytic NPs (e.g., Pt, Pd, Co, Fe, Ni, Cu, Au, Ag, bimetallic NPs, *etc*.) into these structures to exploit their high surface area and interconnected porosity for efficient mass transport in catalytic applications.^22,40,41,73^

### 2.1 Advances in Synthetic Design and Characterization

**Figure 3:**
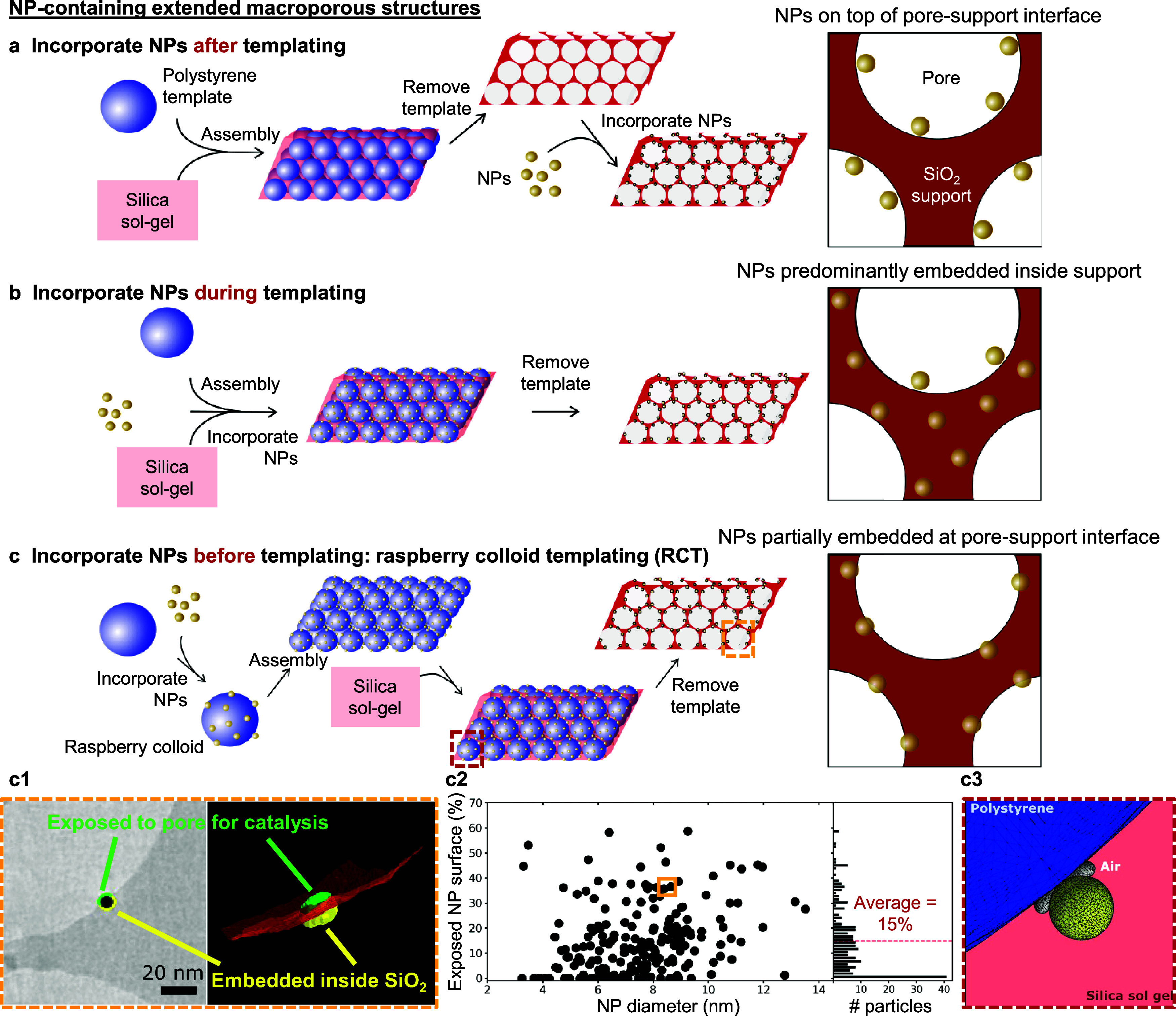
**NP-containing extended 3D macroporous structures.** NPs can be incorporated (a) *after*, (b) *during*, or (c) *before* the self-assembly of templating colloids and infiltration with a support precursor(s) (SiO_2_ sol–gel in these examples). These processes produce NP-containing extended 3D macroporous structures with NPs (a) on the pore surfaces, (b) predominantly fully embedded within the support, or (c) partially embedded at the pore–support interface, as shown schematically by the insets on the right. Yellow and red dashed boxes in (c) are further elaborated in (c1) and (c3), respectively. In raspberry colloid templating (c), (c1) electron tomographic analysis confirms the partial NP embedding (yellow region) within the RCT catalyst support, and (c2) shows a scatter plot of the fractional exposed NP surface area as a function of NP size (left), and as a frequency histogram (right). (c3) Numerical wetting calculations of the steady-state triple-phase contact line at the silica (sol–gel)–NP–polystyrene templating colloid interface reveals the stabilization of an air torus around the NP at the triple-phase interface, resulting in the incomplete wetting of the NP by the SiO_2_ sol–gel and, subsequently, substantial partial embedding of NPs within the formed SiO_2_ support. Data point in the solid yellow box in (c2) describes the NP shown in (c1). (a)–(c) adapted with permission from ref (46). Copyright 2022 ACS (further permissions related to this material should be directed to ACS). (c1)–(c3) adapted with permission from ref (47). Copyright 2021 Wiley-VCH.

Colloidal templating typically forms the catalytic support (Figure 2a), which effectively decouples the NP formation step(s) from support formation. In such colloid-templated structures, NPs can be flexibly incorporated *after, during, or before* colloidal templating (Figures 2a and 3a–c).^46^ Here, we briefly review the characteristics, merits, and limitations in each of the three preparative sequences. Then, we discuss some key advancements in the characterization of these formed structures.

A common approach incorporates NPs *after* the templated structure is fully formed (Figure 3a). Such a templated structure can be regarded as a preformed catalytic support and be subjected to infiltration with preformed colloidal NP solution(s),^71,74,75^ and/or impregnation/precipitation with metal precursor(s),^76−82^ resulting in fully exposed NPs dispersed on the pore surfaces (Figure 3a inset).^46^ This approach is most commonly employed due to the relative ease and scalability of NP incorporation, but is limited by its relatively poor thermomechanical stability. For instance, NP sintering, agglomeration, and/or dislodgement are common deactivation routes for such catalysts under typical thermocatalytic conditions of high temperatures, pressures, and mechanical agitation during catalytic evaluation.^3,72,76,83^

Alternatively, templating colloids can be coassembled with support precursor(s) and NPs (or their metal precursors) *during* colloidal templating (Figure 3b).^84−86^ The inclusion of NPs during templating is uncommon for catalytic applications as the resulting NPs are predominantly fully embedded within the formed support matrix (Figure 3b inset).^84,85,87^ This structural attribute consequently confers a much higher degree of thermomechanical stability (compared to the first approach in Figure 3a), but severely limits NP accessibility to reactants.^72^ Thus, this synthetic strategy is more frequently adopted in photonics^84,87^ and energy storage^85^ applications where NP exposure is not absolutely necessary and desirable NP–support interfacial interactions are maximized through near-complete NP embedding within the support.

A more interesting prospect arises when NPs are incorporated *before* colloidal templating, which we call raspberry colloid templating (RCT, Figure 3c).^22^ In this approach, preformed colloidal NPs are first chemically attached to templating colloids to form NP-decorated colloids termed raspberry colloids.^22^ While inducing NP nucleation on templating colloids is also synthetically feasible,^88^ using preformed colloidal NPs presents an additional advantage of decoupling NP formation from the templating colloid’s surface chemistry,^23^ permitting fully independent preparation of precise and well-controlled designer NPs with specific desirable catalytic attributes (e.g., size, shape, composition, faceting, *etc*.).^44,45^ Following evaporative self-assembly of the raspberry colloids into a colloidal crystal, infiltration with silica sol–gel^23,89^ (noting that other support precursors in the sol–gel^90,91^ or nanocrystal solution^68,69,72^ forms, including mixed support precursors,^92^ are similarly possible), and removal of templating colloids, the final RCT catalyst structure reveals NPs partially embedded at the exposed pore–support interface (Figure 3c inset, 3c1).^22^ Here, we remark that the polymeric templating colloids used in most colloid-templated structures are removed either by calcination,^22,23,47^ or by dissolution and depolymerization using an appropriate solvent mixture.^24,25,69,93^ High temperature calcination typically leaves minimal carbonaceous residue due to the formation of gaseous combustion products but may result in appreciable NP sintering if the NPs are not stabilized within the support, such as when NPs are incorporated *after* colloidal templating.^72^ Alternatively, solvent dissolution can be performed under milder and possibly, ambient conditions, to avoid NP sintering,^24,25,69^ but may leave more carbonaceous residue from any incomplete dissolution of polymeric templates. Another difference between both methods is that calcination enables the complete polycondensation of the sol–gel precursor(s) to form more thermomechanically stable support structures and thus, a mild thermal treatment step is typically incorporated at the end if the solvent dissolution approach is used.^69^ Calcination is preferred for the RCT approach as NP sintering is mostly circumvented by partial NP embedding within the support.^47^ Moreover, apart from removing the polymeric templates, calcination also fully forms the support structure, removes the stabilizing ligands decorating the colloidal NPs to expose the metallic NP surfaces for catalysis,^94,95^ while providing the energy input to access kinetically inaccessible NP shapes and compositions (e.g., alloying of multimetallic NPs and stabilizing specific crystal facets).^23,89,96^

Quantification of NP embedding levels in the RCT catalysts was performed using dual-axis electron tomography which greatly reduced the missing range of angles compared to single-axis electron tomography, thus rendering a more complete 3D reconstruction of the RCT catalyst.^47^ Analysis of more than 200 Au NPs reveals that the mean and median fractional NP surface area exposed was 15% and 12%, respectively, in the SiO_2_ RCT support (Figure 3c2).^47^ In this regard, the partial embedding of practically all NPs using the RCT method takes advantage of the benefits from both earlier approaches: high thermomechanical stability and increased NP–support interfacial interactions (which is achieved by NP incorporation *during* templating, Figure 3b), in combination with the exposure of all NPs for high reactant accessibility (which is achieved by NP incorporation *after* templating, Figure 3a).

The physical origin behind the high degree of NP embedding within the support was elucidated using numerical wetting calculations.^47^ The difference in the wetting contact angle of the polystyrene templating colloid (30°) and the Au NP surface (<10°) to the silica sol–gel resulted in the thermodynamic stabilization of an air-filled torus at the polystyrene–NP–silica sol–gel triple interface, indicative of incomplete NP wetting by the sol–gel which consequently resulted in the partial NP embedding within the formed SiO_2_ support (Figure 3c3).^47^ We anticipate that exploiting surface functionalization to modify the relative difference in the contact angles of the templating colloid and the NP surface to the infiltrating sol–gel may enable even more precise tuning of NP embedding levels (and by extension, NP–support interactions) through adjusting the size of the air-filled torus formed.^47^

Apart from the recent advances on the characterization front in electron tomographic analysis^23,47^ and numerical wetting calculations^47^ of RCT catalysts (Figure 3c1–c3), we point out complementary progress in single-particle tracking of 3DOM structures.^50−53^ Direct and real-time 3D visualization of individual fluorescent trackers diffusing within and between macropores was recently demonstrated using a refractive index matching imaging system.^50,52^ Importantly, the diffusive transport results were readily extended from the single macropore level to the bulk 3DOM structure due to the predictable periodicity and pore interconnectedness in 3DOM structures.^51^ This ease of extrapolating physical properties (e.g., diffusive transport, thermal transport, optical, mechanical) toward the bulk, combined with the high synthetic modularity and thermomechanical stability of RCT catalysts, uniquely positions the RCT method as an excellent model catalyst platform to elucidate clear structure–property relationships in thermocatalysis,^23,89,96,97^ which will be discussed in Section 2.2.

### 2.2 Advantages of Raspberry Colloid Templating (RCT) in Thermocatalysis

**Figure 4:**
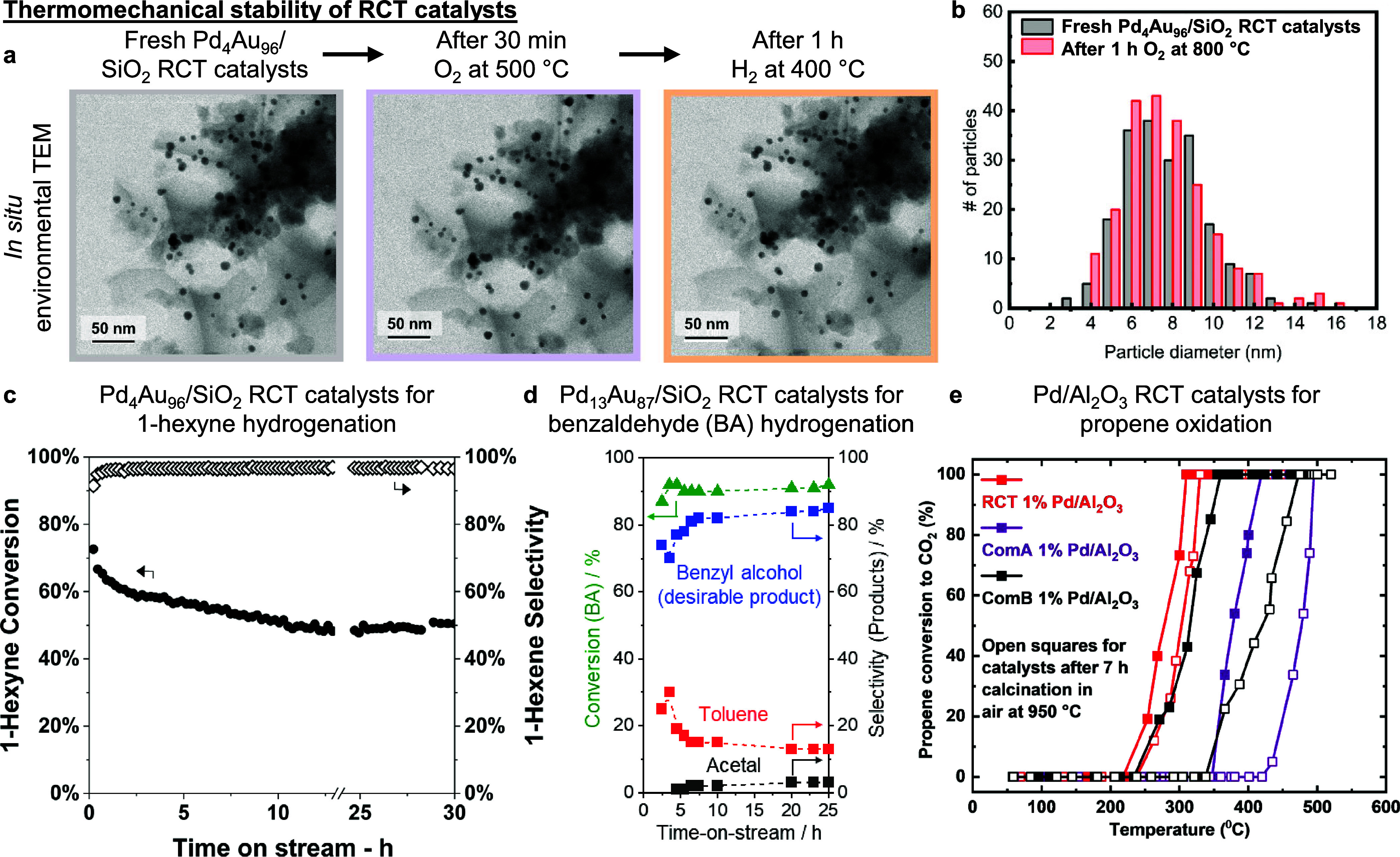
**High thermomechanical stability of RCT catalysts.** (a) *In situ* environmental transmission electron microscopy images depicting the intact macroporous SiO_2_ RCT structure and absence of NP migration after high temperature O_2_ and then H_2_ treatment. (b) Preserved NP size distribution in Pd_4_Au_96_/SiO_2_ RCT catalysts after 1 h of O_2_ treatment at 800 °C, indicating no appreciable NP sintering. The high thermomechanical stability of RCT catalysts allows for the high levels of catalytic activity and selectivity to be maintained in the continuous hydrogenation of (c) 1-hexyne to 1-hexene (gas phase), and (d) benzaldehyde to benzyl alcohol (liquid phase). (e) When tested for propene oxidation, Pd/Al_2_O_3_ RCT catalysts record comparable light-off curves before (filled red squares) and after 7 h of calcination in air at 950 °C (open red squares). For comparison, two different commercial Pd/Al_2_O_3_ catalysts (labeled ComA and ComB) require appreciably higher temperatures to achieve their precalcination conversion levels due to severe NP aggregation and sintering postcalcination. Data of catalysts before and after calcination in (d) are shown with filled and open squares, respectively. (a) adapted with permission from ref (100). Copyright 2020 The Authors under a CC-BY 4.0 license. (b) adapted with permission from ref (47). Copyright 2021 Wiley-VCH. (c) adapted with permission from ref (98). Copyright 2019 American Chemical society. (d) adapted with permission from ref (89). Copyright 2023 American Chemical society. (e) adapted with permission from ref (91). Copyright 2020 Elsevier B.V.

We outline below the advantages of the RCT method from a thermocatalysis application standpoint. The high surface area and interconnected porosity reduce mass transport limitations,^98,99^ while the partial NP embedding resists NP agglomeration, migration, and sintering under thermocatalytic conditions of high temperatures, pressures, and mechanical agitation.^91,100−103^ As a proof of concept, no appreciable changes to the macroporous SiO_2_ RCT support structure and NP size distribution were observed after high temperature O_2_ and H_2_ treatment (Figure 4a–b).^47,100^ The high thermomechanical stability of the RCT catalysts also translates to the preservation of high levels of thermocatalytic activity and selectivity for a broad range of reactions over extended periods of time. Some examples include sustained activity levels when evaluating PdAu/SiO_2_ RCT catalysts for gas-^98^ and liquid-phase^89^ hydrogenation continuously for >24 h (Figure 4c–d). For oxidation, AgAu/SiO_2_ RCT catalysts recorded practically unchanged selectivity to methyl formate in repeated testing across six months without catalyst regeneration,^99^ while Pd/Al_2_O_3_ RCT catalysts retained their propane oxidation activity levels after 7 h of calcination in air at 950 °C, noting that two other commercial Pd/Al_2_O_3_ control catalysts exhibited appreciable deactivation under similar treatment conditions due to NP sintering (Figure 4e).^91^ These promising thermocatalytic stability results using RCT catalysts are in stark contrast to the severe NP leaching and agglomeration observed in Au/TiO_2_ colloid-templated catalysts prepared by incorporating Au NPs *after* TiO_2_ support formation,^72^ emphasizing the critical importance of partial NP embedment within the support structure for stable thermocatalytic applications.

**Figure 5:**
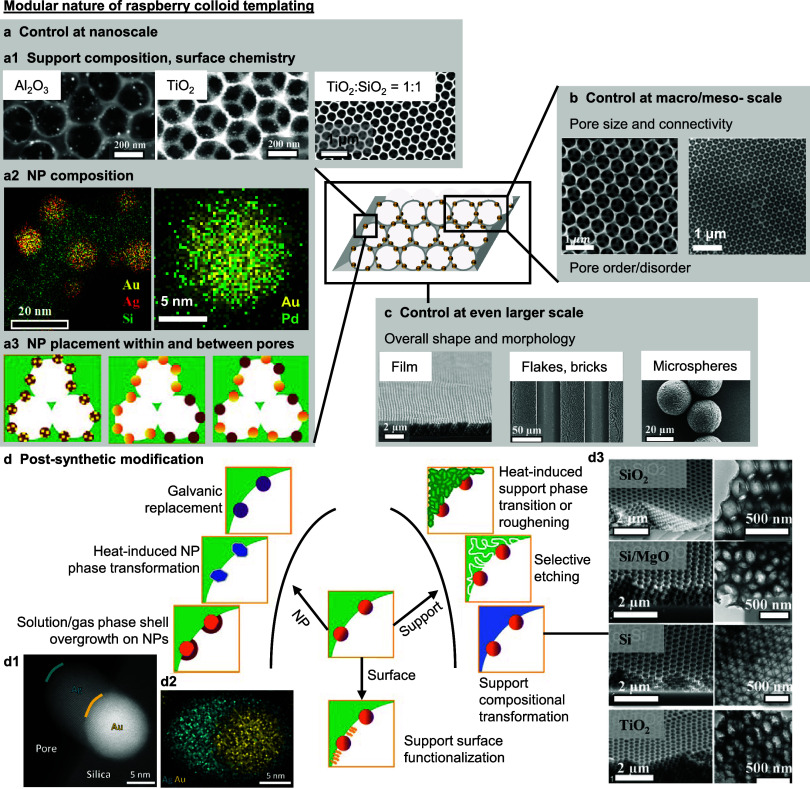
**Modular nature of the RCT catalyst platform.** The modular RCT method affords combinatorial flexibility at different lengths scales: on the (a) nanoscale including (a1) support chemistry, (a2) NP chemistry, (a3) NP placement, (b) macro/meso- scale (pore size, connectivity, ordering), (c) overall shape and morphology. (d) RCT catalysts can also be chemically modified postsynthetically to the NPs, support, and support surfaces. For example, (d1) a second metal (e.g., Ag) can be epitaxially overgrown onto partially embedded Au NPs in Au/SiO_2_ RCT catalysts. Blue and yellow curved lines demarcate the edges of the Ag overgrowth and Au NP, respectively. (d2) Energy dispersive X-ray spectroscopy (EDX) mapping confirms the presence of a thin Ag shell around the embedded sections of the Au NP, suggesting that the embedded segments of the Au NP in the SiO_2_ support of the RCT catalyst are accessible to reactants for catalysis. (d3) The entire support can also be compositionally transformed through postsynthetic surface chemical treatments while preserving the entire 3D ordered macroporous structure. (a1)–(a3), (c), (d) adapted with permission from ref (22). Copyright 2017 Wiley-VCH. (a1) adapted with permission from ref (92). Copyright 2020 Royal Society of Chemistry. (b) adapted with permission from ref (69). Copyright 2021 Wiley-VCH. (d1)–(d2) adapted with permission from ref (47). Copyright 2021 Wiley-VCH. (d3) adapted with permission from ref (67). Copyright 2010 National Academy of Sciences.

Beyond improving stability, the partial NP embedding also increases the NP–support interfacial perimeter and thus, metal–support interactions.^4^ Hence, we expect metal–support interactions to be noticeably enhanced in RCT catalysts as compared to conventional catalysts whereby NPs are nucleated or deposited onto the support surface with markedly smaller NP–support interfacial contact areas. Increasing such interactions is important as metal–support interfacial sites have been reported to serve as new active sites for selective reactant binding^104−106^ and can be even more reactive than both the metal and support sites.^107−109^ Moreover, we have managed to overgrow Ag onto the embedded sections of Au NPs in the RCT catalyst (see Figure 5d2 later),^47^ indicating that even the embedded NP surfaces remain chemically accessible to (some) reactants, possibly due to nanoscale porous channels that form concurrently during support formation when the support sol–gel precursor volume shrinks.^67^ This observation opens possibilities for confined catalysis beyond the catalysis that is taking place in the largely unconfined macropores.^47^

**Figure 6:**
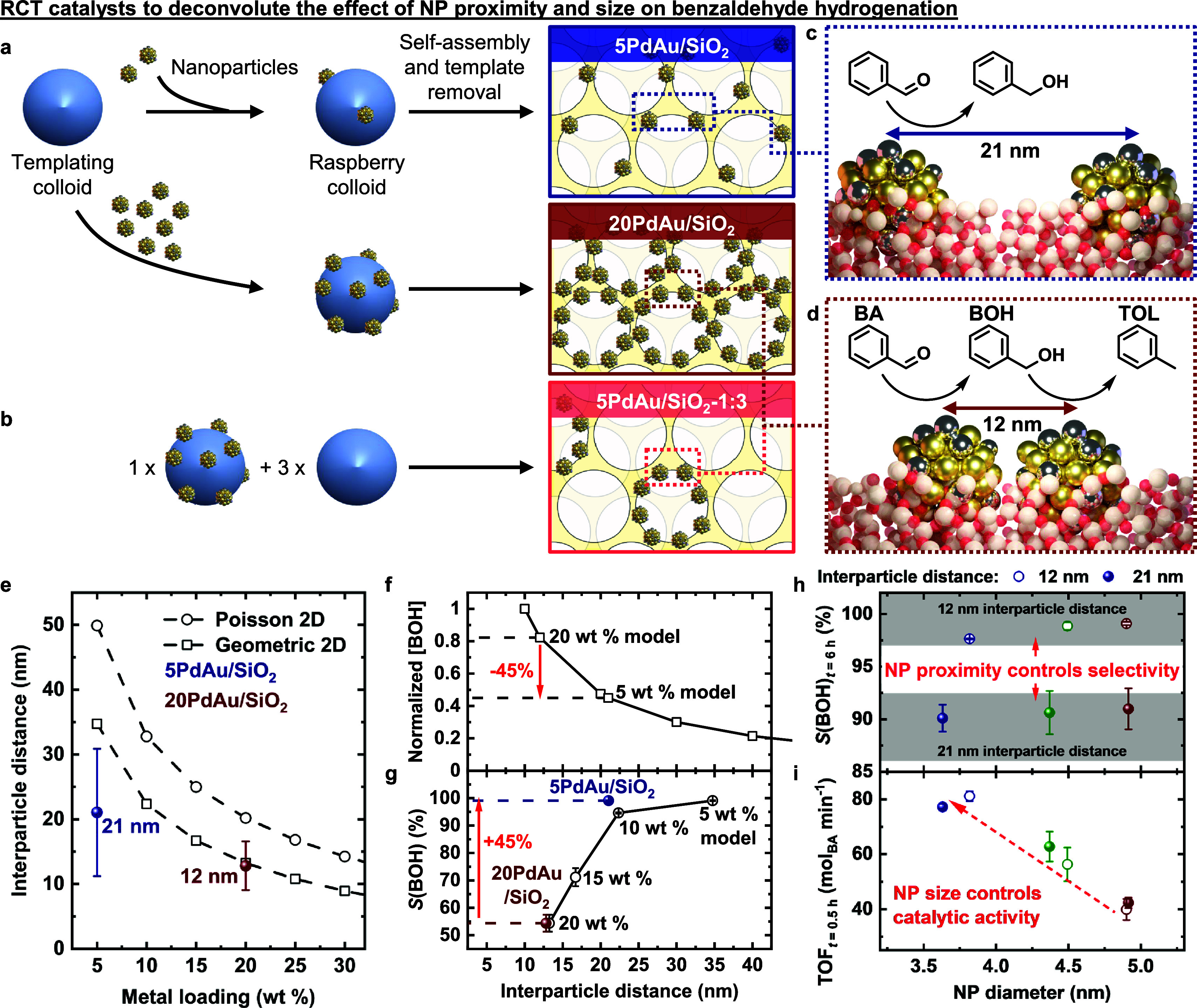
**Effect of NP proximity and size on benzaldehyde hydrogenation using RCT catalysts.** (a) Tuning the average interparticle distance from 21 to 12 nm by increasing the metal loading from 5 to 20 wt %, which in turn is achieved by increasing the NP to templating colloid ratio by a factor of 4 while maintaining the same amount of templating colloids added. *x*PdAu/SiO_2_ refer to *x* wt % Pd_12_Au_88_/SiO_2_ RCT catalysts. (b) To decouple the metal loading from interparticle distance, NP-decorated raspberry colloids (corresponding to 20 wt %) were mixed with NP-free templating colloids in a 1:3 ratio in the self-assembly step to achieve an overall metal loading of 5 wt % for comparison with 5PdAu/SiO_2_ (at the same metal loading) and 20PdAu/SiO_2_ (at the same interparticle distance). Effect of (c) larger and (d) smaller interparticle distance on benzaldehyde hydrogenation. BA, BOH, and TOL refer to benzaldehyde, benzyl alcohol, and toluene, respectively. (e) Average interparticle distance measured experimentally, compared to a 2D statistical (Poisson) and 2D geometrical model. As a function of interparticle distance, (f) COMSOL-simulated local BOH concentration ([BOH]) in between NPs and (g) the experimentally measured selectivity to BOH. As a function of NP size, (h) experimentally measured selectivity to BOH (*S*(BOH)) and (i) turnover frequency (TOF) of benzaldehyde. In (h)–(i), catalysts prepared at larger (21 nm) and smaller (12 nm) interparticle distances are shown with filled and open circles, respectively. (a)–(g) adapted with permission from ref (23). Copyright 2024 Springer Nature. (h)–(i) adapted with permission from ref (96). Copyright 2024 American Chemical Society.

The highly modular nature of the RCT platform also generates a large combinatorial space to independently tailor the catalyst’s properties at different length scales during catalyst synthesis. At the nanoscale, the NP and support chemistry can be tailored by the choice of NP(s) and support precursor(s) used (Figure 5a1–a2). Notably, the RCT method flexibly allows for the assembly of multiple types of NP-loaded and/or NP-free raspberry colloids without affecting the overall catalyst morphology, enabling one to predetermine NP placement within a specific fraction of macropores (Figure 5a3, see also Figure 6b later).^22,23^ This distinct feature of NP localization in the RCT catalysts cannot be achieved when NPs are incorporated during or after templating (Figure 3a–b) as the macropores will be uniformly functionalized in those other approaches. At the macro/meso- scale, the macropore size and connectivity is derived from the size and shape of templating colloids used (Figure 5b). At the overall scale, the final structural morphology is dictated by the combined effects of the colloidal assembly conditions and the underlying patterning surfaces onto which colloidal assembly is performed (Figure 5c).^22,37,110−112^ Following RCT catalyst synthesis, Figure 5d outlines a variety of possible postsynthetic modifications to the bulk chemistry, surface functionality, and structural morphology of both the NPs and support to further expand the structural-compositional space for catalytic applications and beyond.^22^ In particular, we highlight the selective epitaxial overgrowth of a secondary material shell on partially embedded NPs (Figure 5d1–d2) and the complete shape-preserving chemical compositional transformation of the support (Figure 5d3) as two of many possible approaches to access new and more complex NP–support structural–compositional combinations. Importantly, we remark that these postsynthetic modifications simultaneously preserve the most critical structural features of RCT catalysts for thermocatalytic applications: partially embedded NPs in a highly ordered and interconnected 3D macroporous structure with high reactant accessibility.

To put into practice and integrate the various design principles and strategic advantages described earlier, we capitalized on the high modularity and thermomechanical stability of the RCT platform to study the independent effect(s) of NP proximity on the successive hydrogenation of benzaldehyde (BA) to benzyl alcohol (BOH) and toluene (TOL).^23^ This work was motivated by our realization that traditional impregnation and precipitation approaches often do not easily disentangle NP size and proximity effects, both of which are potential catalytic descriptors, from each other. That is, the metal precursor concentration controls the nucleation rate of new NPs, which reduces NP proximity, but simultaneously induces the growth of existing NPs to larger sizes.^10,11^ In this regard, the RCT platform was uniquely suited for this investigation as the interparticle distance can be controlled independently from the NP size through the quantity of preformed NPs loaded (Figure 6a). The partial NP embedding within the support also conferred high thermomechanical stability during catalytic evaluation,^47,91^ permitting the unambiguous investigation of NP proximity effects at a fixed NP size.^23,47^ As alluded to in Figure 5a3, NP-decorated raspberry colloids can also be diluted with NP-free templating colloids in a predefined ratio.^22^ In this example, the interparticle distance was controlled by the NP density on the raspberry colloids used, while the overall metal loading was kept constant by the dilution ratio, which additionally decoupled the interparticle distance from metal loading (Figure 6b).

We reported that catalysts with NPs separated at larger interparticle distances formed less undesired toluene (Figure 6c–d), even when compared at the same overall metal loading and catalyst mass used.^23^ Using electron tomography to determine the exact 3D spatial coordinates of >2000 NPs in the transmission electron microscopy image field-of-view, we calculated the average interparticle distance in the RCT catalysts. The result was in qualitative agreement with purely statistical and geometric models (Figure 6e), indicating that the NPs were well-dispersed in the RCT catalyst, even up to high 20 wt % loadings. We simulated the local concentration profile of the benzyl alcohol intermediate around the NPs and found a clear negative correlation between the benzyl alcohol concentration profile ([BOH], Figure 6f) and selectivity toward benzyl alcohol (*S*(BOH), Figure 6g), both as a function of interparticle distance. The catalyst with the lowest 5 wt % loading and thus, largest interparticle distance, possessed the lowest local BOH concentration around their NPs (Figure 6f), which suppressed BOH readsorption to the same (or adjacent) NP for conversion to toluene, thereby steering selectivity toward BOH (Figure 6c, 6g). The converse scenario using the catalyst with the highest 20 wt % loading (and thus, smallest interparticle distance) also holds true, and its effect is summarized schematically in Figure 6d. This work^23^ demonstrates the unique suitability of the modular RCT platform as a well-defined model catalyst platform to independently isolate and tune potential catalytic descriptors to unambiguously derive structure–property relationships that bridge the materials gap between surface science and technical catalysts.

To further exemplify the ability of the RCT catalyst platform to exert independent control over catalyst design parameters, we designed a set of Pd_12_Au_88_/SiO_2_ RCT catalysts comprising alloyed NPs at three different sizes (3.6, 4.5, and 5 nm) and two distinct interparticle distances (12 and 21 nm).^96^ This study^96^ expanded on the preceding work^23^ and separately examined the isolated effect(s) of NP size at various fixed interparticle distances. Independent of each parameter, we established that NP proximity controls selectivity toward BOH (Figure 6h) while NP size controls catalytic turnover of benzaldehyde (Figure 6i), providing a practical means to maximize BOH yield and overcome archetypical activity–selectivity trade-offs using two descriptors found in virtually all NP-supported catalysts.^96^

We postulate that a possible development to further exploit NP proximity effects will be to prepare RCT catalysts assembled with different raspberry colloids of the same colloidal template size (to maintain a high degree of packing and structural order),^22^ but loaded with different NP loading densities, surface charges, and/or composition. Such chemical variations on the raspberry colloid surface and by extension, the RCT catalyst pore surface, will manipulate the residence time of reactant molecules in specific pores and deliberately enhance or diminish reactant–NP interactions in specific steps of multistep catalytic cascades to direct the overall selectivity toward the desired intermediates or products. Our hypothesis is motivated by recent single-particle tracking efforts in 3DOM structures to derive diffusive transport parameters at the single macropore level.^50−53^ One noteworthy result from those advanced characterization works is that the experimentally measured time taken for the trackers to translocate between adjacent macropores could be orders of magnitude slower than random-walk simulations^50^ due to repulsive electrostatic interactions between the tracker and pore walls.^53^ These results imply that the average residence time of reactant molecules within a macropore is easily altered by microscopic interactions (e.g., electrostatic interactions, adsorption–desorption dynamics) that strongly depend on the chemistry of both the pore surfaces and the reactant(s).^53^ We further anticipate that our hypothesis can also be readily extended to hierarchical macro-mesoporous structures (see Section 3) as their pores can be individually functionalized,^24,25^ and that we can take greater advantage of more hindered transport with decreasing pore sizes in such hierarchically porous systems.^50^

## 3 NP-Containing Hierarchical Macro-Mesoporous Structures

In the 3DOM/IO structure, some naturally occurring secondary meso- and/or micro- porosity may be created in the macropore walls from volume shrinkage associated with the infiltrating sol–gel precursor during the template removal step,^39,67^ or when nanocrystals are randomly packed to form the 3DOM/IO structure.^39,69^ To achieve greater control over the secondary pore structure (i.e., size, placement, connectivity), a second, or even a third template can be intentionally introduced as additional pore-directing agents.^36,38,39,113,114^ Secondary porosity not only creates even more surface area for mass transport, but also unlocks size-selective and confined space catalytic features only found in mesoporous and microporous materials due to their substantially higher frequency of reactant–pore wall interactions in anomalous Knudsen diffusion regimes.^115−118^ While NP-containing hierarchical macro-mesoporous structures are typically prepared by hard colloidal templating for thermocatalytic applications, a combined hard–soft templating approach^119^ presents unique synthetic advantages to achieve spatially disparate active site localization (see Section 3.2).^24,25^

### 3.1 Advances in Synthetic Design

**Figure 7:**
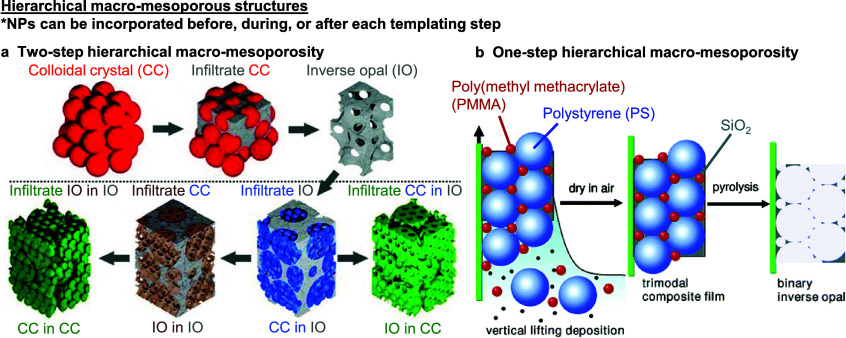
**Hierarchical macro-mesoporous structures.** Hierarchical macro-mesoporosity can be created in (a) two or (b) one step(s). (a) To create hierarchical macro-mesoporosity in two steps, an inverse opal (IO) is first generated, which can be backfilled by mesoscale templates to form a colloidal crystal within the IO (CC in IO). Infiltrating this structure and removing the mesoscale templates yield a mesoporous IO structure within a macroporous IO structure (IO in IO). Backfilling the (IO in IO) or (CC in IO) structure with a support sol–gel precursor and removing all the previous structures yield the inverted (CC in CC) or (IO in CC) macro-mesostructures, respectively. (b) Hierarchical macro-mesoporosity can be created in one step by simultaneous colloidal assembly of both the macro- and meso- scale colloidal templates with the support sol–gel precursor(s) (SiO_2_, in this example) and/or nanocrystals. Note that in both methods, NPs can be incorporated before, during, or after each colloidal templating step, as shown in Figure 3a–c. (a) adapted with permission from ref (120). Copyright 2013 Wiley-VCH. (b) adapted with permission from ref (29). Copyright 2006 American Chemical Society.

Hierarchical macro-mesoporous structures can be fabricated either by introducing a second templating step to a preformed 3DOM/IO structure (Figure 7a),^120−122^ or by a three-component coassembly approach to simultaneously create both pore structures (Figure 7b).^29,123,124^ The former allows the macropore structure to be independently functionalized prior to the infiltration of mesoscale templating colloids and in principle, can form four combinatorially distinct hierarchical structures as shown in Figure 7a.^120^ The latter is more convenient but one will need to further consider and modulate both the type and magnitude of surface interactions between the macroscale and mesoscale colloidal templates to avoid phase separation that would otherwise compromise the coassembly process.^37,125^ Regardless of the approach taken, the additional complexity in creating hierarchical macro-mesoporosity can more easily lead to disordered structures^126,127^ that do not substantially affect their thermocatalytic applications, but will pose additional challenges to characterization^128^ and modeling.^129^ We propose that for such hierarchical macro-mesoporous structures to benefit from both levels of porosity (i.e., macro: absence of diffusion limitations; meso: confined catalysis; both: active site compartmentalization, directed reactant flow), such structures should be fabricated in ways that create interconnected macro-mesoporosity for full accessibility to both pore systems.

Principles for NP incorporation into 3DOM structures described in Section 2.1 similarly apply to hierarchical macro-mesoporous structures:^46^ NPs can be incorporated either as metal precursors and/or colloidal NPs before, during, or after each colloid templating step (Figure 2a, 3a–c). We emphasize from Section 2.1 that the reactant accessibility and thermomechanical stability of the NPs to resist sintering under thermocatalytic conditions is contingent on the extent of NP embedding within the support. Here, the main distinguishing characteristic of hierarchically macro-mesoporous structures (as compared to the macroporous structures described in Section 2) is that they possess even greater synthetic flexibility for spatially disparate NP incorporation, which has implications in multistep catalytic cascades that require multiple active sites, precise NP placement, and directed reactant flow to maximize catalytic selectivity and avoid antagonistic side-reactions (see Section 3.2).^24,25^

### 3.2 NP-Containing Extended Hierarchical Macro-Mesoporous Structures in Thermocatalysis

Spatially disparate active site functionalization of hierarchical macro-mesoporous structures can be synthetically tricky as most hierarchical colloidal crystals are typically formed using differently sized colloidal templates of similar chemistries for convenient dispersion, self-assembly, and template removal. For instance in Figure 7b, the colloidal templates are both organic polymeric beads of polystyrene and poly(methyl methacrylate), which implies that most chemical and/or heat treatments to remove any one type of template will also most likely remove the other template type too.^29^ Consequently, any NP or surface functionalization on the macropore surface will likely also be applied to the mesopores and vice versa, thus not achieving the desired spatially disparate NP or active site functionalization property.

**Figure 8:**
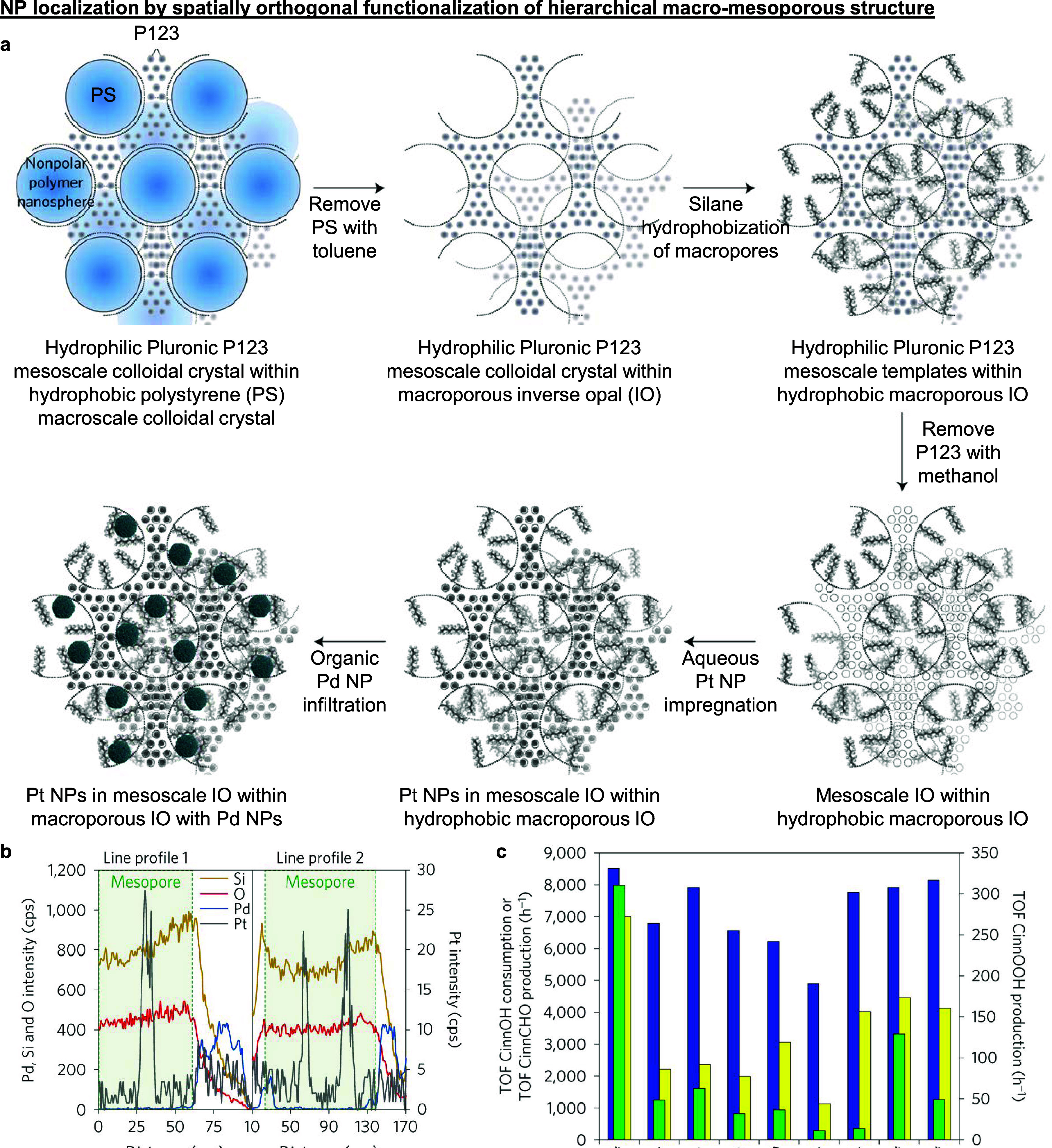
**NP localization by spatially orthogonal functionalization of extended hierarchical macro-mesoporous structures.** (a) A hierarchical hydrophobic–hydrophilic macro-mesoporous structure was sequentially incorporated with NPs, first by impregnating 2.4 nm Pt NPs in the aqueous phase within the hydrophilic mesopores, followed by the immobilization of nonpolar oleylamine-stabilized 5.6 nm preformed colloidal Pd NPs within the hydrophobic macropores. (b) Energy-dispersive X-ray spectroscopy (EDX) line scans across the hierarchical macro-mesoporous structure confirms disparate Pt and Pd NP localization in the mesopores and macropores, respectively. (c) Rate of cinnamyl alcohol consumption (blue) to produce cinnamaldehyde (yellow), and then cinnamic acid (green), over a variety of structural controls to illustrate the importance of NP placement and controlling reactant flow through the catalyst structure. (a)–(c) adapted with permission from ref (25). Copyright 2016 Macmillan Publishers.

One elegant synthetic advance reports an extended hierarchically ordered macro-mesoporous silica structure using a combined hard–soft templating approach described schematically in Figure 8a.^25^ A binary colloidal crystal comprising larger hydrophobic polystyrene colloids with hydrophilic Pluronic P123 mesoscale colloids packed in its interstitial gaps was fabricated. The very different polarities of the polystyrene and P123 colloids enabled the stepwise removal of each colloidal template type by sequential dissolution with toluene and then methanol. Importantly, the authors hydrophobically silanized the macropores before methanol exposure, which produced a hierarchical hydrophobic–hydrophilic macro-mesoporous structure. Exploiting the vastly different pore surface polarities, the hierarchical structure was first selectively impregnated with aqueous Pt precursors (forming Pt NPs) at the hydrophilic mesopores, then infiltrated with preformed Pd NPs (dispersed in an organic phase) at the hydrophobic macropores, successfully localizing Pd and Pt NPs in the macropores and mesopores, respectively (Figure 8b). We remark that a highly packed and ordered binary colloidal crystal structure is critical in ensuring that solvents can easily access and dissolve away all the templating colloids to create the intended interconnected macro-mesoporosity for high reactant accessibility. We stress the authors’ use of both surface polarity and NP size differences as key success factors to achieve effective NP localization. Specifically, aqueous impregnation fills only the small hydrophilic mesopores and not the hydrophobic macropores, then infiltration with preformed and hydrophobically functionalized 5.6 nm colloidal Pd NPs decorated the large 350 nm macropores. We note that the Pd NPs were intentionally synthesized larger than the 3.5 nm mesopores to prevent their entry into mesopores.

**Figure 9:**
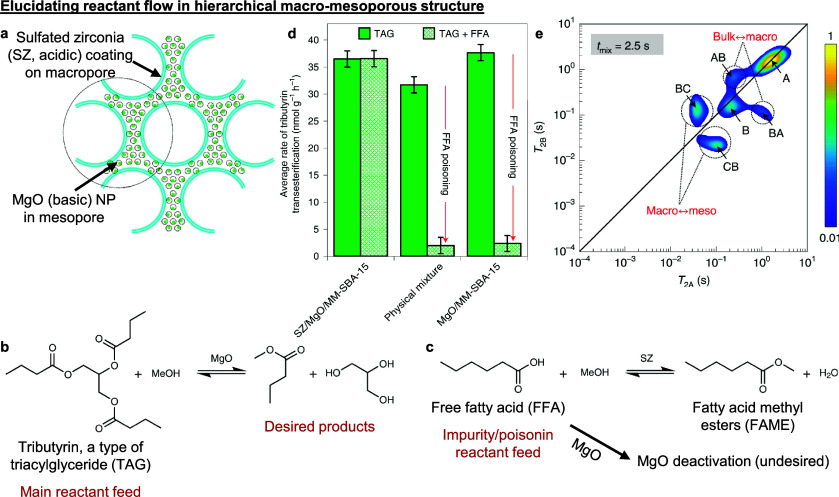
**Spatially disparate acid–base active site localization for catalytic cascades with antagonistic side-reactions.** (a) A hierarchical acid–base macro-mesoporous structure was produced by sulfation treatment of zirconia-treated macroporous surfaces, followed by basic MgO NP nucleation in the mesopores. (b) MgO base-catalyzed transesterification of TAG. (c) Sulfated zirconia acid-catalyzed esterification of FFA impurities to benign FAME. FFA rapidly poisons the basic MgO active sites and needs to be removed from the TAG reactant feed for high catalytic conversion of TAG. (d) Rate of tributyrin (a type of TAG) transesterification in (from left to right) the hierarchical acid–base structure, a physical mixture of the acid-only and base-only catalyst, and the base-only catalyst. Error bars represent standard error from the mean (*n* = 3). (e) NMR relaxation-exchange correlation experiments confirm bulk ↔ macropore ↔ mesopore transport in the hierarchical structure. Bulk, macropores, and mesopores are labeled as sites A, B, and C, respectively. Off-diagonal peaks confirm diffusion between the pair of sites listed in the plot within the first 2.5 s mixing time. Peak intensities are indicated by the color bar (linearly scaled). (a)–(e) adapted with permission from ref (24). Copyright 2020 Springer Nature.

When the hierarchical macro-mesoporous catalyst was evaluated in the sequential oxidation of cinnamyl alcohol to cinnamaldehyde and then to cinnamic acid, achieving high yields of cinnamic acid required fulfilling two conditions.^25^ First, bifunctionality (i.e., both Pd and Pt) was essential, as Pd and Pt NPs were most active for the first and second oxidation steps, respectively. Second, control over NP placement and reactant flow was critical to ensure high selectivity toward cinnamic acid as Pd and Pt NPs promote undesired side reactions involving cinnamaldehyde and cinnamyl alcohol, respectively. This point was reinforced by the low cinnamic acid yields in the other control structures where both types of NPs decorated both pores (Figure 8c). This finding implies that cinnamyl alcohol first flows from the bulk through Pd NP-decorated macropores to achieve near-full conversion to cinnamaldehyde, following which cinnamaldehyde diffuses into Pt NP-decorated mesopores to be converted to cinnamic acid, avoiding side reaction(s) that would have otherwise occurred if the reactant flow or NP localization was reversed. This proposed direction of reactant transport was indeed confirmed in a follow-up work (see Figure 9e later).^24^

To unambiguously verify the direction of reactant flow within such hierarchical macro-mesoporous structures, the same group fabricated a similar structure whereby the macropores were now functionalized with an acidic sulfated zirconia coating, while the mesopores were decorated with basic MgO NPs (Figure 9a).^24^ In the base-catalyzed transesterification of triacylglycerides (TAG) (Figure 9b), the challenge was to prevent acidic free fatty acid (FFA) impurities in the TAG feed from poisoning the basic MgO sites in the mesopores. When a combined feed of TAG and FFA was used, only the hierarchical acid–base macro-mesoporous structure allowed FFA to be first converted to neutral fatty acid methyl esters (FAME) at the acidic macropores (Figure 9c) before the benign FAME, together with the rest of the TAG feed, diffused into the mesopores for TAG transesterification. On the other hand, the base MgO catalyst alone or a physical mixture of the acid and base catalyst were both extremely susceptible to FFA poisoning at the basic sites (Figure 9d), highlighting the importance of controlling both the reactant flow and active site placement, especially in reactions with antagonistic side-reactions. Crucially, NMR relaxation-exchange correlation experiments unequivocally verified that reactant transport occurred from the bulk ↔ macropore ↔ mesopore, with no evidence for direct bulk ↔ mesopore diffusion (Figure 9e). We point out that this noninvasive technique is highly suitable as an *ex situ* probe to quantify diffusive transport^48,49^ and adsorption phenomena,^130^ even in optically opaque porous materials (i.e., most catalytic supports). We thus encourage further adoption of this and other transport measurement techniques to complement existing computational fluid dynamics modeling.^131,132^

## 4 NP-Containing Discrete Hollow Nanoreactors

Apart from the extended macroporous catalyst structures derived from templating a self-assembled colloidal crystal (Figure 2a, Sections 2 and 3), individual colloidal particles of various shapes can also act as standalone templates for material overgrowth as shells (e.g., carbonaceous materials,^133^ zeolites,^20^ metal oxides/chalcogenides,^18,19,134^ including their doped derivatives, Figure 2b).^28^ Subsequent removal of the template reveals a single hollow chamber delimited by at least one secondary material shell, with the whole entity known as a hollow nanoreactor.^18^ Unlike extended macroporous structures that template an extended 3D colloidal crystal, working with a discrete colloidal template presents additional synthetic flexibility to devise even more unique anisotropic structures and morphologies using surface, sol–gel, and interfacial wet chemistry (e.g., yolk@shell, multishelled, onion-/urchin-/cage-/tube-/dendritic-like structures *etc*.).^18^ The shell(s) can also be treated postsynthetically to introduce secondary porosity (meso-/micro-) and/or surface textures.^19^ We remark that the physical features of the hollow nanoreactor (e.g., morphology, aspect ratio, porosity, and thickness of its shell(s)) play a substantial role in the overall thermomechanical stability of such hollow nanoreactors. Thus, we encourage future designs to incorporate features that not only enhance catalytic activity and selectivity, but also catalytic stability.

Like the extended macroporous structures described in Section 2.1, NPs can similarly be flexibly loaded at different stages of the templating process (before, during, or after; Figure 2b). In hollow nanoreactor structures, the wider morphological variety in the hollow cavity and (multiple) shell structure(s) opens even greater possibilities for NP incorporation and placement control. For instance, NPs can be incorporated either on or embedded (partially or fully) in the internal and/or external shell surfaces, within mesopore channels in the shell(s), or localized at specific shell locations in asymmetrically grown shell structures.^18^ We separately note that enzymes have been reliably immobilized together with NPs to yield hybrid heterogeneous-biocatalytic systems^135,136^ for specialty chemical synthesis.^137^ Given the multitude of combinatorial possibilities, we concisely highlight three creative recent examples in colloid-templated hollow nanoreactor design that not only compartmentalize active sites and dictate the direction of reactant flow, but also synergize the differently isolated active sites to enhance catalytic performance. We refer readers to a few comprehensive reviews^18,135,138−140^ focused solely on hollow nanoreactors design principles for completeness.

### 4.1 NP-Containing Discrete Hollow Nanoreactors in Thermocatalysis

**Figure 10:**
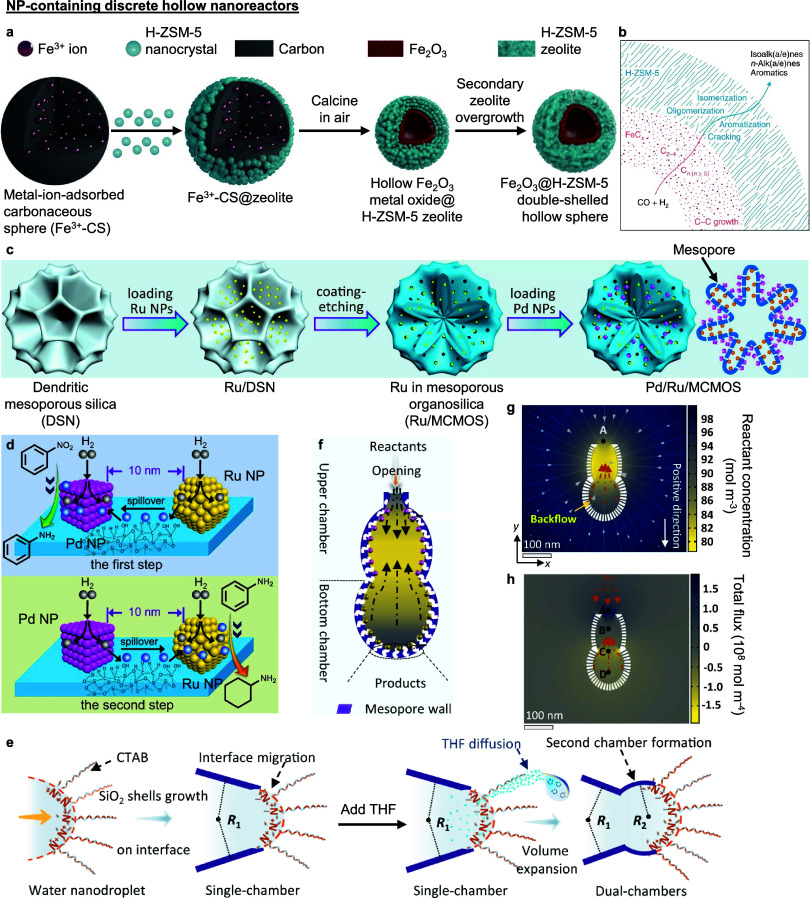
**NP-containing discrete hollow nanoreactors.** (a) Synthesis of Fe_2_O_3_@H-ZSM-5 metal oxide@zeolite double-shelled hollow sphere. (b) Direction of reactant flow (outward) and sequence of reactions in the double-shelled hollow sphere during Fischer–Tropsch synthesis. (c) Synthesis of mesoporous organosilica nanoreactor with Ru and Pd NPs localized on the interior and exterior walls of the nanoreactor, respectively. (d) Close proximity between Ru and Pd NPs (∼10 nm) facilitates H spillover across the mesopore channel to assist in the sequential hydrogenation of nitrobenzene to aniline (top) by Pd NPs, and then aniline to cyclohexylamine (bottom) by Ru NPs. (e) Nanodroplet remodeling strategy to produce hierarchical multichambered mesoporous silica nanoreactors. SiO_2_ walls are shown in purple. (f) Directed reactant flow (black arrows) within a two-chambered mesoporous silica nanoreactor. (g) Time-dependent simulated reactant concentration profile, and (h) corresponding reactant flux and flow (orange arrows) in the two-chambered nanoreactor at 20 μs. (a)–(b) adapted with permission from ref (20). Copyright 2022 Springer Nature. (c)–(d) adapted with permission from ref (19). Copyright 2021 The Authors under a CC-BY 4.0 license. (e)–(h) adapted with permission from ref (21). Copyright 2022 The Authors under a CC-BY 4.0 license.

Fischer–Tropsch synthesis for long hydrocarbon chain production involves carbon chain growth of the CO reactant, followed by structural transformation of the carbon backbone. These reactions are catalyzed by metallic and zeolitic acidic sites, respectively, which was efficiently achieved using a Fe_2_O_3_@H-ZSM-5 double-shelled hollow structure (Figure 10a).^20^ Zeolite NP seeds were first nucleated on an Fe^3+^-doped carbonaceous colloidal template in a high ionic strength solution mixture to reduce double-layer repulsion between both components. Calcination then removed the carbonaceous template and solidified the Fe_2_O_3_ inner shell. A second zeolitic growth step densified the outer zeolite NP shell and restored colloidal stability. The mechanism of shell crystallization (inward) and hollow cavitation was elucidated by *in situ* Raman spectroscopy and X-ray diffraction. When applied to Fischer–Tropsch synthesis, the highest selectivity to C_5_–C_11_ products was recorded using the double-shelled structure, as compared to other structural controls. This result was due to (1) adequate formation of FeC_*x*_ (from Fe_2_O_3_ shell reduction during reaction) that promoted carbon chain growth, (2) intimate contact between the Fe metal and zeolite acidic sites in adjoined shells that facilitated a seamless cascade reaction through a predetermined reactant flow pathway (Figure 10b), and (3) the hollow cavity elevating the local concentration of both reactants and intermediates to accelerate conversion.^20^ Notably, the double-shelled structure maintained high levels of CO conversion and product selectivity for 45 h time-on-stream, which the authors attributed to the thicker zeolitic shell preventing the catalyst structure's physical/mechanical disintegration during operation.

While most hollow nanoreactors enable precise control over NP placement and can effectively direct reactant flow, a recent work provides compelling evidence that distinct NPs, when compartmentalized in different locations but at short proximity to each other, can interact with each other to synergistically enhance the catalytic performance of both NP types.^19^ Here, the hollow nanoreactor is a multicompartment mesoporous organosilica structure with Ru and Pd NPs decorating the interior and exterior shell surfaces, respectively. Ru NPs were impregnated on dendritic mesoporous silica template surfaces, after which a layer of mesoporous organosilica was overgrown around the entire structure before removing the original dendritic template and immobilizing colloidal Pd NPs on the exterior surface (Figure 10c). In our opinion, the most interesting observation was the enhanced catalytic activity in both sequential hydrogenation steps of nitrobenzene to aniline, and aniline to cyclohexylamine. Even though both types of NPs were spatially isolated from each other by mesopore channels, the close ∼10 nm proximity between them facilitated non-negligible hydrogen spillover from the other NP type not participating in either hydrogenation steps (Figure 10d top and bottom, respectively), which was unequivocally verified by H_2_ temperature-programmed reduction and kinetic analyses. This work demonstrates that with proper templating strategies, active sites can not only be spatially isolated from each other to avoid antagonistic side reactions and active site deactivation (as illustrated in Figures 8 and 9), but can still be brought into sufficiently close proximity with each other to enhance the activity in each step of the catalytic cascade without noticeable detriment to overall catalytic selectivity and stability. We point out that this nanoreactor structure was initially stable but experienced slight Ru NP leaching and sintering after six catalytic reuse cycles, which we posit can be improved by partially embedding the NPs into the organosilica support during the nanoreactor design and synthesis.

We finally highlight a successive soft template remodelling strategy that exploits surface and interfacial chemistry to gain precise control over the number of adjoined chambers in the nanoreactor structure (Figure 10e).^21^ In brief, a porous SiO_2_ shell was grown around a cetyltrimethylammonium bromide (CTAB)-stabilized water nanodroplet (template) in pentanol, which caused the droplet–solvent interface to migrate outward as SiO_2_ was partially grown around the droplet. Addition of tetrahydrofuran (THF) and its diffusion into the water droplet expanded the nonrigid side of the droplet to create a smaller adjoined second chamber as SiO_2_ continued to form. In principle, this remodelling strategy can be used to create one new chamber with each successive THF addition step, albeit at increasingly slower rates due to the decreasing differential in THF concentrations inside and outside the droplet. Importantly, different NPs can be incorporated at each chamber synthesis step, which was exploited to improve selectivity for a selective hydrogenation catalytic cascade with reactants flowing from the outer to the inner chamber of a two-chambered nanoreactor (Figure 10f). Using finite element analysis, the simulated transient reactant concentration profile revealed rapid depletion of reactants at the upper (outer) porous chamber where the first hydrogenation step occurred (yellow region in Figure 10g), causing more reactants to diffuse from the bulk solution into the upper outer chamber (A to B in Figure 10h). This rapid depletion of reactants (via conversion to intermediates) in the upper chamber also created a concentration gradient for reactants to diffuse from the lower (inner) chamber to the upper chamber (D to C in Figure 10h). This example illustrates the relevance of colloidal templating as an integral strategy to devise asymmetric hollow nanoreactors to realize directed reactant flow and induce substrate channelling effects,^17^ which will only continue to grow with future advancements in synthetic methodology of colloidal templates as material building blocks.^141,142^

## 5 Conclusions and Perspective

This Perspective seeks to bring to attention colloidal templating as a viable synthetic strategy that can be incorporated into catalyst design for thermocatalysis through three broad structures of interest: extended 3D macroporous structures, extended hierarchical macro-mesoporous structures, and discrete hollow nanoreactors. The key synthetic principles, advantages, potential challenges, and opportunities for each of these colloid-templated structures are summarized in Table 1. We remark that colloidal templating does not replace, but instead, complements traditional impregnation and precipitation methods in catalyst design to facilitate more complex and/or well-defined catalytic investigations.

**Table 1:** Summary of the Different Colloid-Templated Structures for Thermocatalysis

NP-containing structure	Key synthetic principles	Advantages and applications	Potential challenges and opportunities
Extended 3D macroporous structures (Section 2)	NPs, preformed and/or nucleated *in situ*, are incorporated at different stages (*before*, *during*, or *after*) of the colloidal templating step(s) to fabricate the macroporous support structure	NP and support formation steps are entirely decoupled from each other for independent tuning and optimization at different length scales	Choice of NP incorporation *before*, *during*, or *after* colloidal templating step(s) has a pronounced impact on the final NP stability and accessibility (Section 2.1)
		Well-defined and interconnected porosity facilitates investigation into physical transport properties, and the extrapolation of such results from the single pore level to the macroscopic level	RCT method (NP incorporation *during* colloidal templating) provides high thermomechanical stability and accessibility due to partial NP embedding within the support matrix (Section 2.2)

Extended hierarchical macro-mesoporous structures (Section 3)	Secondary mesoporous structure is created with, or after, the primary macroporous structure is formed	Hierarchical macro-mesoporosity creates additional surface areas for mass transport of reactants and products	Creating such complex structures necessitates spatially independent template removal and pore functionalization steps, which inadvertently increases synthetic complexity (Section 3.1)
	NPs, preformed and/or nucleated *in situ*, are incorporated *before*, *during*, or *after* each colloidal templating step(s)	Mesoporosity unlocks size- and shape- selective catalytic properties found only in anomalous and confined Knudsen diffusion regimes, whereas unconfined catalysis can occur on large surface areas of macropores	With orthogonally functionalized templating colloids and solvents (e.g., polar and nonpolar), together with judicious NP size selection, spatially disparate active site functionalization and consequently, compartmentalization, have been achieved (Section 3.2)
		Reactant transport is predefined in such hierarchical porous structures (bulk ↔ macropores ↔ mesopores), which can be exploited for selective catalytic cascades	

Discrete hollow nanoreactors (Section 4)	Individual colloidal particles of various shapes as standalone sacrificial templates for additional material shell growth	Standalone template affords the greatest synthetic flexibility among all three structures: anisotropic and precisely tailored templates have been exploited to compartmentalize active sites and prescribe reactant flow within nanoreactor structures	The design challenge (and opportunity) is to not only compartmentalize incompatible active sites, but to also synergize the different active sites (via engineering active site proximity) to achieve catalytic outcomes beyond the sum of its individual parts (Section 4.1)
	NPs can be incorporated *before*, *during*, or *after* each colloidal templating step(s)		

In NP-containing extended 3D macroporous structures (Section 2.1), we evaluated the implications of NP inclusion *before*, *during*, or *after* colloidal templating on the resulting NP accessibility and overall catalytic stability (Figure 3a–c).^46^ We highlight the RCT method whereby NPs are incorporated *before* the colloidal templating step (Figure 3c) to yield partially embedded NPs within the macroporous support structure (Figure 3c1–c2) in Section 2.2.^22,47^ The RCT method produces catalysts with high thermomechanical stability by virtue of partial NP embedding (Figure 4), together with high reactant accessibility to the NPs,^47^ which represent the key advantages conferred by NP incorporation *during* and *after* colloidal templating, respectively. The full decoupling of the NP and support formation steps advantageously allows for designer NPs of precisely specific compositions (*e.g*. multimetallic, high entropy) and morphologies (e.g., core–shell, random alloy, patchy, shapes exposing specific facets) to be incorporated into the catalyst structure, that would otherwise be synthetically challenging to nucleate on the support surfaces using conventional impregnation and precipitation. As an illustration, we describe how RCT catalysts can be modularly designed to attain independent control over catalytic descriptors at various length scales (Figure 5).^22,47^ This synthetic flexibility facilitated the unambiguous investigation of NP proximity (as a collective NP property) without concomitant changes to other catalytic descriptors (Figure 6), which cannot be easily achieved synthetically in catalysts prepared solely using conventional impregnation and/or precipitation approaches.^23^

We next discussed how to create secondary mesoporosity in 3D macroporous structures to produce NP-containing extended hierarchical macro-mesoporous structures (Figure 7) in Section 3.1.^29,120^ Such hierarchical structures exploit the high surface area of macropores for mass transport, together with confined diffusion and size-selective properties in the adjacent connected mesopores.^115−118^ In Section 3.2, we discussed how a hierarchical macro-mesoporous structure prescribes a well-defined bulk ↔ macropore ↔ mesopore direction of reactant flow which, when combined with spatially disparate active site placement/functionalization in the different interconnected pore systems, affords highly selective catalytic cascades (Figure 8) capable of circumventing antagonistic side reactions (Figure 9).^24,25^

Thereafter, we assessed synthetic advances in hollow nanoreactors comprising a hollow cavity delimited by material shell(s) and containing catalytically relevant NPs. Such discrete structures are derived from a single colloidal template and flexibly provide a substantially wider compositional–morphological space for the resulting shell(s) and NP incorporation.^18^ In Section 4.1, we highlight hollow nanoreactor designs that combine synthetic expertise in colloidal, sol–gel, surface, and interfacial chemistry to not only compartmentalize the different active sites for highly selective cascade-type transformations and achieve substrate channelling effects,^17^ but also keeps these dissimilar sites in sufficiently close proximity to synergistically enhance the catalytic performance of each active site through spillover effects (Figure 10).^18−21^

Interspersed throughout this Perspective, we also highlight important advances in characterizing these colloid-templated porous catalyst structures. Electron tomography, especially when using dual tilt axes, enables a wide-angle reconstruction to pinpoint the exact 3D spatial coordinates for large numbers of NPs,^7,23,47^ even within highly textured and porous catalytic structures, to quantify structural parameters such as the average interparticle distance (Figure 6e)^23^ and NP embedding levels (Figure 3c2).^47^ To track reactant flow within porous structures, NMR relaxation-correlation^24,48,49^ is particularly useful for catalytic supports that are optically opaque and unsuitable for optical characterization (Figure 9e). Developments in single particle tracking allow for the experimental determination of a tracker’s residence time within macropores and to visualize their translocation between macropores.^50−53^ Employing these advanced characterization tools has derived many new fundamental insights, some of which were previously predicted by computational modeling of reactant flow. Hence, we encourage continued development and adoption of advanced characterization tools to further understand the catalyst structure and their interactions with reactants to refine catalyst design and enhance catalytic performance.

Our outlook on the future roles, opportunities, and anticipated challenges of colloidal templating in catalyst design for thermocatalysis are as follows. First, we highlight the unique suitability of highly ordered 3D macroporous and hierarchical macro-mesoporous structures as ideal model catalysts to study fundamental catalytic and transport phenomena. Their periodic structure, interconnected porosity (i.e., no dead ends for reactants), and well-defined pore geometry allows characterization and modeling results to be reliably extrapolated from the single pore level to the entire extended structure with high fidelity.^51^ These structures are unlike other structurally inhomogeneous catalyst structures which may not reasonably allow for the extrapolation of characterization data from a narrow sample/region of the catalyst (e.g., electron microscopy of a single flake is often assumed to be representative of the catalyst, but may not hold true in some instances). Moreover, the high synthetic modularity of these structures affords independent control over different structural properties (i.e., potential catalytic descriptors), rendering them suitably positioned to bridge the materials gap between surface science and technical catalysts.^23,96^ Specifically for thermocatalysis, we emphasize that thermomechanical stability is a key prerequisite to preserve all other physicochemical characteristics except the parameter in question during catalytic evaluation. Such thermocatalytically stable catalysts will enable us to unambiguously establish clear structure–property trends without being confounded by concomitant changes in other potential descriptors, which we exemplify in detail using the RCT catalysts in Section 2.2.^23,96^ The encouraging laboratory-scale stability results of such colloid-templated catalysts^20,23,47,89,91,96^ provide a good starting point to evaluate them under industrially relevant conditions (e.g., Fischer–Tropsch synthesis) for benchmarking against technical catalysts prepared by traditional nontemplating methods.

Second, while we center our discussions on thermocatalytic applications, we acknowledge that the structures discussed herein are already adopted in other closely related applications such as photonics, optics, sensing, and photo/electro/photoelectro- catalysis.^26,41^ Efforts should be made to integrate new knowledge acquired from these application fields (and beyond) to refine catalyst design by colloidal templating,^46^ in concert with new advances in traditional catalyst preparative methods.^3^ As an example, our group used the knowledge gained from pore re-entrant geometry and liquid infiltration into inverse opal structures for sensing applications^31,143−145^ to elucidate why NPs were partially embedded when incorporated using the RCT method (Figure 3c3).^47^

Third, we noticed that colloidal templating methods often entail the multistep preparation of complex, highly modular, and sometimes, hierarchical, catalyst structures. We therefore anticipate that future colloid-templated catalyst designs will build on the integration, self-assembly, and modularity capabilities of these prevailing designs. We emphasize that modularity in catalyst design allows one to strategically design catalysts with specific properties and functions that cannot be easily achieved using traditional catalyst preparative methods.^22^ In this regard, we look forward to future catalyst designs that comprehensively consider the role of each material component, their effective integration with each other, and take even greater advantage of the catalyst structure at different length scales from the nano-, micro-, meso-, and to the overall macroscopic catalyst structure.^20^ More importantly, as corroborated by many others in the field, we should look for potentially synergistic or antagonistic effects between material components and strive toward cohesive colloid-templated catalyst designs that outperforms the sum of its individual material components.^19−21,24,25^

Lastly, we remark that widespread adoption of the colloid-templated catalyst structures described in this Perspective, whether at the laboratory bench or test bed scales, can only be encouraged by reducing, streamlining, and/or automating the multiple labor-intensive synthetic steps involved in fabricating such intricate structures. Such efforts necessitate further exploration into colloidal, sol–gel, surface, and interfacial chemistry, together with self-assembly concepts, to parallelize synthesis and achieve high synthetic throughput of these colloid-templated catalysts. To this effort, we identify the use of premade and industrially accessible templates as a critical factor to accelerate the technological readiness of colloid-templated catalysts toward practical and broad-based implementation. We predict that high surface area fibrous templating scaffolds,^146^ such as carbon nanotubes, metal oxide nanofibers, and organic lignin/cellulose/biomass-derived nanofibers, can be colloidally dispersed and surface functionalized for NP incorporation, then backfilled with a (or multiple) metal oxide precursor(s) before selectively removing the templating scaffolds (by combustion, etching, and/or solvent dissolution) to yield highly interconnected NP-containing porous catalyst structures at scale.^90,147^ Such nanofibers, whether polymeric^148−150^ or inorganic,^151^ can be produced at larger scales using electrospinning^152^ or other spray drying approaches,^146^ with NP incorporation already being demonstrated during^148,149^ or after^150,151^ the nanofibers are spun. Toward this endeavor, we foresee that the fundamental insights and design principles gained from the well-defined colloid-templated structures described in this Perspective and in other closely related works will beneficially guide cost-effective and synthetically viable scale-ups for industrial adoption to complement most other industrially relevant catalysts prepared by traditional impregnation and precipitation methods.
